# Determination of Silver(I) by Differential Pulse Voltammetry Using a Glassy Carbon Electrode Modified with Synthesized *N*-(2-Aminoethyl)-4,4′-Bipyridine

**DOI:** 10.3390/s101211340

**Published:** 2010-12-13

**Authors:** Maria-Cristina Radulescu, Ana Chira, Medeea Radulescu, Bogdan Bucur, Madalina Petruta Bucur, Gabriel Lucian Radu

**Affiliations:** 1 Centre of Bioanalysis, National Institute of Research and Development for Biological Sciences, 296, Splaiul Independentei, Bucharest 060031, Romania; E-Mails: ana_chira@yahoo.com (A.C.); bucurica@yahoo.com (B.B.); madalina_dondoi@yahoo.com (M.P.B.); 2 Faculty of Applied Chemistry and Materials Science, Politehnica University of Bucharest, 1-7, Polizu, Bucharest 011061, Romania; E-Mail: rglucian2000@yahoo.com (G.L.R.); 3 Faculty of Chemistry, Department of Analytical Chemistry, University of Bucharest, Sos. Panduri No. 90, Sect. 5, Bucharest 050663, Romania; E-Mail: medeea_radulescu@yahoo.com (M.R.)

**Keywords:** differential pulse voltammetry, diazonium salt, modified glassy carbon electrode, silver, *N*-(2-aminoethyl)-4,4′-bipyridine

## Abstract

A new modified glassy carbon electrode (GCE) based on a synthesized *N*-(2-aminoethyl)-4,4′-bipyridine (ABP) was developed for the determination of Ag(I) by differential pulse voltammetry (DPV). ABP was covalently immobilized on GC electrodes surface using 4-nitrobenzendiazonium (4-NBD) and glutaraldehyde (GA). The Ag(I) ions were preconcentrated by chemical interaction with bipyridine under a negative potential (−0.6 V); then the reduced ions were oxidized by differential pulse voltammetry and a peak was observed at 0.34 V. The calibration curve was linear in the concentration range from 0.05 μM to 1 μM Ag(I) with a detection limit of 0.025 μM and RSD = 3.6%, for 0.4 μM Ag(I). The presence of several common ions in more than 125-fold excess had no effect on the determination of Ag(I). The developed sensor was applied to the determination of Ag(I) in water samples using a standard addition method.

## 1. Introduction

Silver ions or colloidal/nanoparticles are used in a large number of applications in the photographical industry, electrochemistry, medicine and are included in different household products for marketing purposes due to their antiseptic properties. All forms of silver are highly toxic [1–3]. Silver is found in various waste-streams, creating pollution problems due to its aquatic toxicity [4,5]. Some of the silver ions pass through the municipal sewage treatment plants into surface waters while the rest is incorporated into the biosolids.

Sensitive analytical methods are required for the reliable measurement of Ag(I) due to its typical low concentration levels in different real samples (environmental and waste waters, foods, *etc*). Because the reduction potential of Ag(I) is extremely positive (about 0.8 V), this analyte cannot be directly determined by polarography due to sample interferences [6]. Only a limited number of articles have been published on the use of chemically modified glassy carbon electrodes for Ag(I) determination. Some of the used modifiers are: polyaniline [7], *p*-*tert*-butylthiacalixarene [8], chitosan [9] and polypyrrole film [10].

Different 4,4′-bipyridine derivatives have been used as electron relays in horseradish peroxidase biosensors [11] and are attractive compounds for electrode modification used in the detection of heavy metal ions [12]. As potential ligands, 4,4′-bipyridine derivatives are particularly interesting because these species are electroactive and their structures can bridge between metal centres to give coordination polymers [13]. They exhibit fast reversible electrochemical responses at negative potentials, high electron transfer efficiency and low cost, which makes them useful as ligands or redox mediators for numerous reactions.

Electrochemical reduction of different diazonium aromatic derivatives was characterized and used for surface modification of different carbon electrodes (glassy carbon, graphite, carbon fiber, carbon paste, carbon nanotubes, *etc.*) in various practical applications [14–19]. Diazonium modified electrodes are stable for a long time in air and resistant to sonication in nonpolar organic solvents [20,21]. The stability of these electrodes and versatility of the modification method with diazonium salts are especially attractive characteristics for stripping analysis of metals [22–25].

**Scheme 1.**
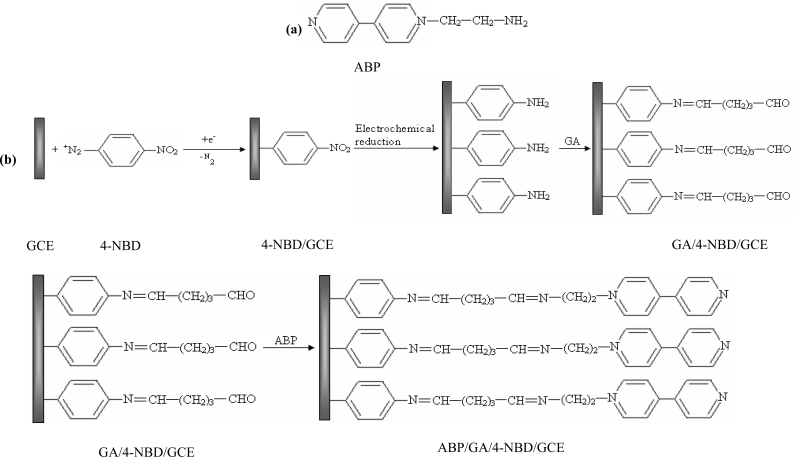
**(a)** Structure of ABP. **(b)** Attachment of ABP to the GA/4-NBD/GCE.

The aim of our study was to develop a method to modify GCE surface using a synthesized monoaminated alkyl derivative of 4,4′-bipyridine for the electrochemical determination of Ag(I). For this purpose we have modified 4,4′-bipyridine with an amino moiety in order to bind it on the electrode surface by crosslinking with glutaraldehyde. First, 4-nitrobenzene groups were grafted on the electrode by electrochemical reduction of the corresponding diazonium salt. In the next step, the nitro groups from the GCE surface were reduced to amino groups by applying a catodic potential. Subsequently, the amino groups from the GCE surface were activated with glutaraldehyde (GA) [26] and finally the *N*-(2-aminoethyl)-4,4′-bipyridine (ABP) was bound to the active sites of the GA (Scheme 1). The obtained electrode was used for Ag(I) preconcentration and determination by differential pulse voltammetry in different water samples.

## 2. Experimental Section

### 2.1. Reagents

*N*-(2-aminoethyl)-4,4′-bipyridine (ABP) was synthesized from 4,4′-bipyridine and 2-chloro- ethylamine (both reagents from Acros Organics, www.acros.com). All other reagents: acetonitrile (ACN, for HPLC), tetrabutylammonium tetrafluoroborate (TBA), 4-nitrobenzendiazonium tetrafluoroborate (4-NBD), potassium ferrocyanide, glutaraldehyde (GA) 25%, potassium phosphate monobasic, sodium phosphate dibasic, potassium chloride, sodium acetate, acetic acid, hydrochloric acid, nitric acid, Ag(I) nitrate were from Sigma-Aldrich (www.sigmaaldrich.com). Aqueous solutions were prepared with purified water (18 MΩ cm^−1^, Millipore, USA, www.millipore.com/). A stock solution of 1.0 × 10^−3^ mol/L AgNO_3_ was prepared with distilled water and stored in the dark. The Ag(I) standard solutions were prepared daily by dilution of the stock solution. The buffers used were: acetate (0.1 M sodium acetate, 0.1 M acetic acid, pH 5.2, 0.1 M KCl) for analysis, citrate (0.05 M sodium citrate, 0.05 M citric acid and 0.1 M KCl, pH 3.0) for glutaraldehyde washing and phosphate buffer saline PBS (0.06 M Na_2_HPO_4_, 0.04 M KH_2_PO_4_ and 0.1 M KCl, pH 7.0) for electrode characterization by cyclic voltammetry and for glutaraldehyde reaction.

### 2.2. Apparatus

All electrochemical measurements were performed using a PGSTAT302N potentiostat/galvanostat (Metrohm-Autolab, The Netherlands, www.metrohm-autolab.com) equipped with three-electrode cell (Metrohm) and controlled using Nova 1.5 software. The working electrode was a 3 mm in diameter GCE from Metrohm, reference electrode was an Ag/AgCl//3M KCl (Metrohm) and counterelectrode was a Pt wire. An Autolab pX1000 module was used for pH corrections. The stripping measurements were carried out in deoxygenated solutions under pure nitrogen atmosphere at room temperature.

### 2.3. Synthesis of *N*-(2-Aminoethyl)-4,4′-Bipyridine (ABP)

ABP was synthesized according to a literature procedure [27] by refluxing a solution containing 0.078 g of 4,4′-bipyridine and 0.058 g of 2-cloroethylamine (a 1:1 molar ratio) in 30 mL of acetonitrile and water (1:1) for 10 hours. The reaction product was analyzed by HPLC.

### 2.4. Glassy Carbon Electrode (GCE) Modification

The procedure for modifying the GCE surface is based on the reduction of diazonium salt and was adapted from a previous protocol developed, optimized and characterized by our group for enzyme immobilization on platinum (micro)electrodes [26]. The GCE was modified with ABP in four independent steps following Scheme 1: (1) electrografting of diazonium salt on GCE surface; (2) electrochemical reduction of nitro groups to amino groups; (3) functionalization of electrode with glutaraldehyde (GA); (4) covalent attachment of *N*-(2-aminoethyl)-4,4′-bipyridine (ABP). GCE surface was polished with alumina (0.3 μm, Metrohm, England), thoroughly rinsed with Milli-Q water and dried with a nitrogen gas stream. Then the surface was grafted with 4-nitrophenyl groups by using a solution of 5 mM 4-NBD and 50 mM TBA (as supporting electrolyte) in acetonitrile. This process was performed by chronopotentiometry for 30 s at −0.25 μA. After that, the electrode was washed with acetonitrile, distilled water and dried with a nitrogen gas stream. The reduction of nitro groups to amino was made in a PBS solution pH 7 by chronoamperometry for 60 s at −0.5 V. The amino groups from GCE surface were activated with GA vapors (20 μL GA 25% in a sealed 5 mL Berzelius beaker) for one hour. The electrode was sonicated in PBS for 15 min to remove the unreacted GA from the surface. Covalent binding of ABP on GA was performed by adding onto the electrode surface 20 μL of 1 mM ABP solution in PBS and allowing it to react an hour at room temperature. In the end the modified GCE was thoroughly washed again with large amounts of water to remove the unreacted bipyridine derivative.

### 2.5. Ag(I) Analysis Method

A preconditioning of the modified GCE surface was carried out before each analysis by recording ten cyclic voltammograms from −0.2 to +0.6 V at a scan rate of 50 mV/s in the electrolyte solution (acetate buffer). The modified GCE was immersed in 25 mL Ag(I) standard or sample stirred solution buffered at pH 5.2. Ag(I) was reduced to Ag(0) by applying a potential of −0.6 V for 180 s followed by 15 s equilibration. Then, the electrode was moved to another electrochemical cell which contained a deoxygenated electrolyte solution of pH 5.2 and DPV was recorded from +0.1 to +0.5 V with 50 mV/s scan rate; 100 mV pulse amplitude; 4 ms modulation time and 25 mV step potential. After the analysis, the modified GCE was cleaned at 0.3 V for 60 s in an acetate buffer solution to remove completely the accumulated analyte. One modified GCE can be used more than 30 times.

## 3. Results and Discussion

### 3.1. Characterization of GCE Modified with ABP

**Figure 1.**
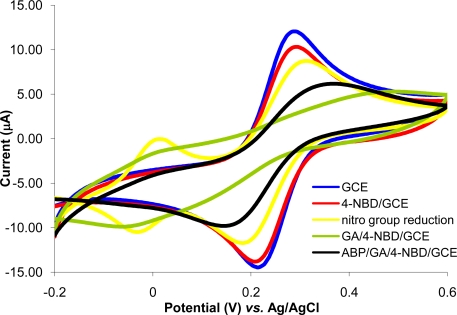
The cyclic voltammograms of 1 mM Fe(CN)_6_^3−^ in PBS (pH 7, 0.1 M KCl) on a polished GCE ( 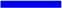); 4-NBD/GCE ( 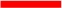); after reduction of nitro groups to amino ( 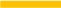); GA/4-NBD/GCE ( 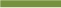); ABP/GA/4-NBD/GCE ( 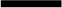).

The immobilization protocol of ABP on GCE is based on the electrode surface modification with 4-NBD. Amino groups used for ABP crosslinking with glutaraldehyde are bounded on GCE surface. This method was previously optimized and characterized for acetylcholinesterase immobilization on Pt (micro)electrodes [26]. Each step of the diazonium modification of Pt (micro)electrodes was characterized using cyclic voltammetry and electrochemical impedance spectrometry at two pH values (3.0 and 7.0) in order to modify the electric charge of different functional groups from electrode surface [26]. It was demonstrated that, it is possible to monitor the deposition of each layer, the type of moieties found on electrode surface and to establish the optimum working conditions that allow electrode modification with minimum surface fouling by using the peak intensity in CV and Rct in EIS. For the experiments reported in this paper, each step of GCE modification was characterized by cyclic voltammetry using as redox probe a solution of 1 mM potassium ferricyanide in PBS (pH 7.0 in 0.1 KCl) at 0.1 V/s scan rate in the domain of −0.2 to +0.6 V. The voltammograms recorded for 1 mM potassium ferricyanide in PBS are shown in Figure 1. Experiments were performed in parallel with polished GC electrodes to establish if any of the compounds was adsorbed on electrode surface. The nonspecific adsorption problem was eliminated by respecting the optimum conditions.

The reversible peaks of Fe(CN)_6_^3−^ observed with polished GCE were slightly reduced at the GA/4-NBD/GCE and also at the ABP/GA/4-NBD modified GCE, but the electron transfer still takes place at a significant rate. This demonstrates the possibility to use the electrode and only partial surface fouling is produced by modification. During the optimization of electrode modification protocol the minimization of surface fouling was a key factor taken into consideration.

A nonspecific adsorption was observed during GA contact with the surface of diazonium modified GCE. Activation with GA of amino groups from electrode surface (step three of the modification protocol) was studied using different methods: (1) by immersing the electrode in solutions with different concentrations of GA in PBS pH 7.0; (2) by deposing a drop of 10 μL GA solution on the GCE surface and (3) by keeping the GCE in the GA vapors atmosphere. The use of GA in solution leads to surface passivation while the reaction using the GA vapors atmosphere minimized this problem. After the reaction with GA the electrode was sonicated for 15 min in PBS pH 7.0, citrate buffer pH 3.0 or water, to remove the unreacted GA. The best results were obtained by sonication in PBS. Cyclic voltammograms of 1 mM Fe(CN) _6_^3−^ in PBS were also recorded with polished GCE and after the immersing of GCE in GA vapors atmosphere for one hour. It was observed that the peaks of Fe(CN) _6_^3−^ ions were smaller than those obtained with polished GCE due to a small adsorption of GA on electrode surface. The GA/GCE was then sonicated in PBS for 15 min and the adsorbed GA was removed (Supplementary Material Figure S-1).

Nonspecific adsorption of the ABP on the surface of polished GCE was studied by immersing the GCE in a solution of 1 mM ABP in PBS for 2 h, 4 h and 20 h and recording the cyclic voltammograms of 1 mM Fe(CN) _6_^3−^ in PBS. No significant changes were observed even after 20 h suggesting the absence of nonspecific adsorption of ABP on the surface of the GCE (Supplementary Material Figure S-2). Nonspecific adsorption of ABP was also absent on a GCE modified with 4-NBD, but without the GA activation step. The same studies were made using 2-chloroethylamine and again it was not observed any significant nonspecific adsorption.

The storage stability of the GCE modified with ABP was studied in different conditions: in PBS pH 7.0, citrate buffer pH 3.0, water or in air. After 1 h, 20 h and 3 days no significant modification of cyclic voltammograms of 1 mM Fe(CN) _6_^3−^ in PBS was observed when the electrodes were kept in PBS and for this reason the modified GCE must be stored before and after use in PBS.

DPV determination of Ag(I) was performed with 3 different blank electrodes in order to justify the usefulness of our synthesized 4,4′-bipyridine derivative: bare GCE, 4-NBD modified GCE and GCE/NBD/GA 2-chloroethylamine. A solution of 1 μM Ag(I) was used for these experiments. For the bare GCE it was obtained only a small and wide peak. In the case of a GCE modified only with 4-NBD no peak was found from 0.1 V to 0.5 V. When a 4-NBD/GCE modified with 2-chloroethylamine was used, the recorded DPV for a blank solution showed only an increased signal noise. No peak was identified from 0.1 V to 0.5 V for 1 μM Ag(I). These results indicate that the accumulation and DPV analysis of Ag(I) ions on the surface of the modified GCE was a consequence of the complexing and electrochemical mediator properties of the ABP.

### 3.2. The Influence of the Supporting Electrolyte in the Stripping Medium

The intensity of DPV peaks is strongly influenced by the working conditions. Among experimental parameters that one has to optimize are: pH value and composition of the supporting electrolyte, the deposition time, reduction potential and stripping scan rate. The first parameters investigated were the pH and the composition of the supporting electrolyte. Different solutions were tested: 0.1 M HNO_3_, 0.1 M HCl, acetate buffer pH = 3.6–6.5, 0.2 M KNO_3_, PBS (pH 7; 0.06 M Na_2_HPO_4_, 0.04 M KH_2_PO_4_). The results had shown that the voltammetric peaks corresponding to the Ag(0) oxidation were obtained in all cases. The best analytical signal was obtained using a deoxygenated solution of acetate buffer pH 5.2. The composition of the supporting electrolyte may produce the precipitation or complexation of the silver ions formed by stripping. This may have a positive effect on the analytical performances of voltammetric techniques, like the Br^−^ that produces soluble AgBr_2_^−^ after AgBr precipitation [28].

### 3.3. Reduction Potential

The reduction potential, accumulation time and scan rate were optimized one variable at the time. The effect of the reduction potential on the intensity current of the analytical signal was studied by varying the reduction potential from 0 to −0.8 V for a concentration of 0.4 μM Ag(I) in 0.1 M acetate buffer pH 5.2 and a reduction time of 30 sec. The highest peak current was obtained at −0.6 V, while smaller analytical signals were obtained for the other tested potentials (Supplementary Material Figure S-3).

### 3.4. The Effect of the Accumulation Time on the Analytical Signal

The dependence of anodic peak current on Ag(I) accumulation time was investigated using a concentration of 0.4 μM Ag(I) for accumulation times between 30 s and 240 s. The peak current was found to significantly increase with the accumulation time until 180 s, followed by a plateau. For maximum sensitivity, a 180 s accumulation period was used for all the measurements (Supplementary Material Figure S-4).

### 3.5. Scan Rate

DPV has the advantage that it increases S/N ratio due to minimization of capacitive current. The influence of scan rate on the anodic peak current was investigated in the 0.001–0.1 V/s interval. It was observed a linear increase of anodic peak current with the scan rate between 0.001 V/s and 0.05 V/s, a behavior characteristic for adsorbed electroactive substances. For scan rates higher than 0.05 V/s Ag(I) peaks were wider and in consequence anodic peak current decreased. Hence, a scan rate of 0.05 V/s was deemed optimum (Supplementary Material Figure S-5).

### 3.6. Calibration Curve, Detection Limit and Reproducibility

**Figure 2.**
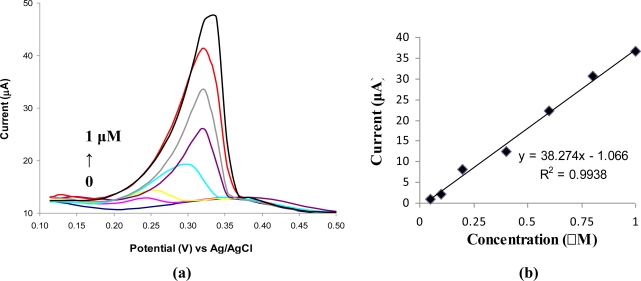
**(a)** Differential pulse voltammograms of Ag(I) accumulated on the ABP-modified GCE. Ag(I) concentrations were: 0 μM, 0.05 μM, 0.1 μM, 0.2 μM, 0.4 μM, 0.6 μM, 0.8 μM, 1 μM. **(b)** The calibration graph.

The DPV voltammograms recorded for different concentrations of Ag(I) prepared in acetate buffer solution (0.1 M, pH 5.2) are presented in Figure 2a. A linear calibration graph was obtained in the concentration range of 0.05 μM to 1 μM Ag(I) with the equation (Figure 2b):

$$\text{I}(\mu \text{A})=38.274\times \text{Conc}.\;{\text{Ag}}^{+}(\mu \text{M})-1.066\;\;\;\;\;({\text{R}}^{2}=0.9938,\;\;\text{n}=7)$$

Electrode surface saturation was observed at the values of concentrations higher than 1 μM Ag(I). The detection limit was estimated to be 0.025 μM Ag(I) for 180 s of accumulation time. The relative standard deviations for six successive measurements of 0.1 μM Ag(I) and 0.4 μM Ag(I) were 4.7% and 3.6%, respectively. The sensibility of the modified electrodes is 5.4 A/(M*mm^2^) and was calculated from the slope of the calibration graph divided by the geometric area of GCE (7.06 mm^2^).

This limit of detection obtained with the proposed modified electrode for Ag(I) detection is better than those obtained with other modified electrodes. The limits of detection reported in literature for silver analysis was 4.8 mM for an electrode modified with *p*-isopropylcalix[6]arene [28], 0.2 mM for a carbon paste electrode modified with S_2_O_2_-donor [29], 0.063 mM for a vermiculite modified carbon paste electrode [30] and 0.5 mM for an carbon paste electrode [31].

### 3.7. Study of Interferences

Potential interference from several common ions with Ag(I) analytical signal was investigated. The modified GCE was immersed in a electrochemical cell containing the support electrolyte, 0.8 μM Ag(I) and different concentrations of Cu(II), Zn(II), Cd(II), Pb(II), Fe(III) or Ni(II). After accumulation, the DPV scan was performed in the same domain, from −0.2 V to +0.6 V. Only a peak corresponding to Ag(0) oxidation was observed in the presence of the tested ions. No interferences were observed in the presence of 0.1 mM Zn(II), Pb(II), Fe(III), Ni(II). In the presence of 0.1 mM Cu(II) and Cd(II) was observed a 11.5% and 6% decrease in the analytical signal of Ag(I). The results show that the modified GCE is selective and can be used for Ag(I) determination in water samples without any significant influence from other common metal ions.

### 3.8. Real Sample Analysis

The ABP-modified GCE was used for the determination of Ag(I) in water samples. The samples were collected from the Dambovita River in downtown Bucharest and from the tap water from the Bucharest public network. Five hundred mL of each sample was filtered and buffered with sodium acetate/acetic acid 0.1 M. 25 mL of each solution were added in the electrochemical cell and the working procedure was applied. Because the presence of the Ag(I) in the real samples was not detected the standard addition method was applied under the same experimental conditions. The analytical results and recoveries for Ag(I) ions added to the sample solutions are given in Table 1. The recoveries of Ag(I) ions added to the sample solutions were between 96%–106%.

## 4. Conclusions

ABP was synthesized to introduce an amino moiety in a 4,4′-bipyridine structure. This amino group was used for attaching the compound to a GCE surface using 4-NBD based chemistry. The obtained modified GCE is stable and can be regenerated for at least 30 successive analyses. A DPV analysis method specific for Ag(I) detection was developed by modification of a GCE with ABP. The developed modified GCE presents good sensitivity and reproducibility and a great selectivity for Ag(I) determination. The analyte ion is preconcentrated by chemical interaction with the ABP-modified GCE. The calibration graph of Ag(I) showed a linear dependence from 0.05 μM to 1 μM Ag(I), with a sensibility of 38.327 μA/μM and a detection limit of 0.025 μM Ag(I). No significant or only minimum interferences from other cations were noticed at interfering concentration levels 125 times higher than that of the analyte.

## Supplementary Information



## Figures and Tables

**Table 1.** Recovery results of Ag(I) added into tap water and river-sample (n = 4 ± SD).

**Samples**	**Added (μM)**	**Found (μM)**	**Recovery (%)**
Tap water	0.2 0.4 0.6	0.192 ± 0.01 0.419 ± 0.02 0.616 ± 0.02	96 105 103
River-water	0.2 0.4 0.6	0.210 ± 0.01 0.425 ± 0.01 0.613 ± 0.03	105 106 102
