# Supplementary material for: Determination of Silver(I) by Differential Pulse Voltammetry Using a Glassy Carbon Electrode Modified with Synthesized N-(2-Aminoethyl)-4,4′-Bipyridine
Source: Sensors (Basel). 2010 Dec 13;10(12):11340–51. doi: 10.3390/s101211340 (PMC3231042; doi:10.3390/s101211340)

Supplementary Information

Figure S1. Cyclic voltammograms of 1 mM $Fe(CN)_6^{3-}$ in PBS buffer (pH 7, 0.1 M KCl) recorded at: polished GCE (—); GA/GCE (—); GA/GCE after sonication (—).

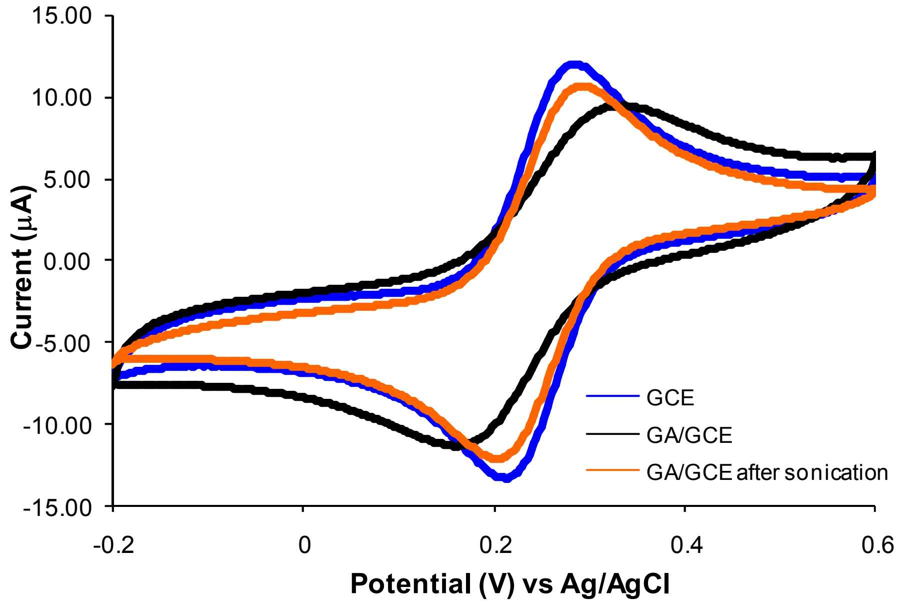

Figure S2. Cyclic voltammograms of 1 mM $Fe(CN)_6^{3-}$ in PBS buffer (pH 7, 0.1 M KCl) recorded at polished GCE (—) and at polished GCE after immersion in a solution of ABP for 2 h (—), 4 h (—) and 20 h (—).

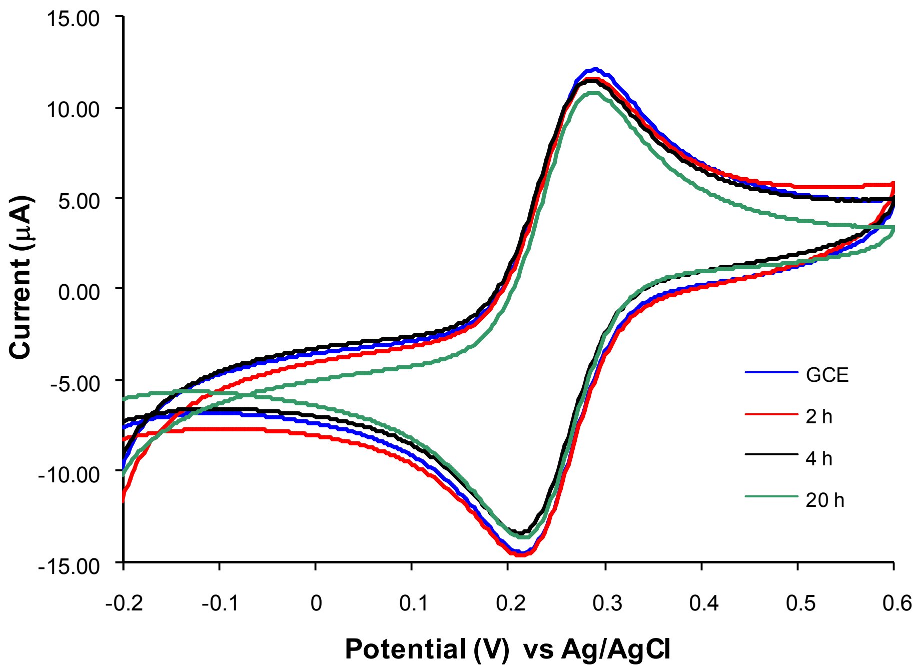

Figure S3. The effect of the reduction potential on the analytical signal of 0.4 μM Ag(I). The reduction potentials were: −0.1 V; −0.2 V; −0.4 V; −0.6 V; −0.8 V.

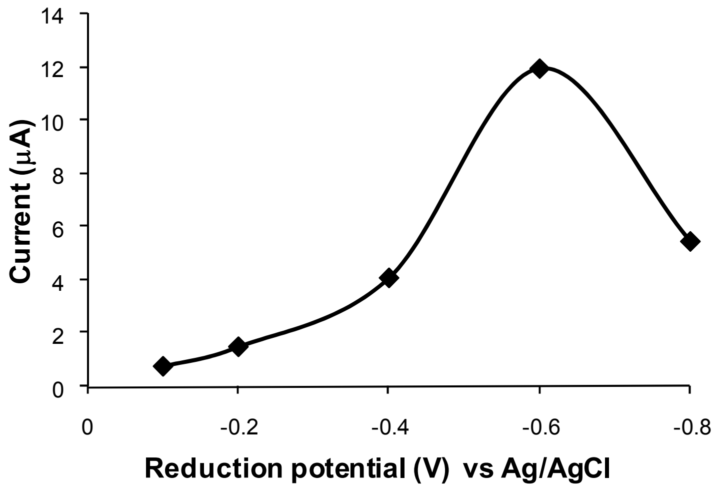

Figure. S4. The effect of the reduction and accumulation time on the analytical signal of 0.4 μM Ag(I). The times were 30 s; 60 s; 90 s; 120 s; 180 s; 210 s; respectively 240 s.

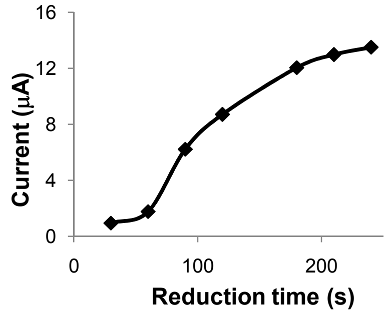

Figure S5. The effect of the scan rate on the analytical signal of 0.4 μM Ag(I). The domain of the studied scan rate was from 0.001 V/s to 0.1 V/s.

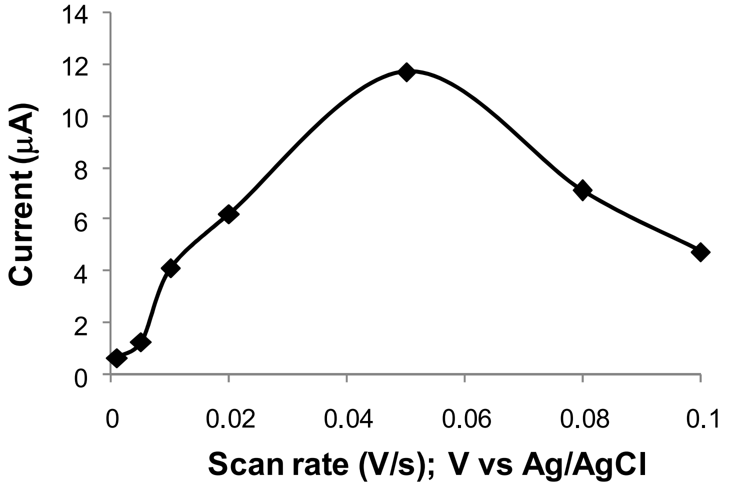

Supplement: Supplementary file 1 [file sensors-10-11340-s001.pdf]
